# The Artificial and Natural Ventilation of Hospitals

**Published:** 1887-04-30

**Authors:** Keith D. Young


### The Artificial and Natural Ventilation of Hospitals.

By Keith D. Young, F.R.I.B.A.

The subject treated by Dr. Theodore Williams in his admirable paper is one which is peculiarly interesting to me as an architect concerned in hospital work. The Hospital for Consumption occupies, very naturally, a large share of Dr. Williams' attention; and the conclusions he draws in favour of artificial ventilation are, I infer, largely based on the experience gained from the working of the system at Brompton. I venture, however, to think that so far as general hospitals are concerned these conclusions are hardly borne out by past history. The only other hospital that I know of in London where artificial ventilation is in use is Guy's, where in the new wing two large towers form the air-duct to all the wards, and the extraction-shaft is a third and higher tower—the impulsion of air being attained by fans aided by heat, and the expulsion by heat. These wards were certainly at one time condemned by the medical staff as the most unhealthy in the hospital.

Instances could be given of many hospitals where systems of artificial ventilation have been abandoned, as either positively injurious or of no definite advantage. The former case applies to the Bristol General Hospital, York County Hospital, and Edinburgh Royal Infirmary, and the latter to St. Mary's Hospital, Paddington, and the London Fever Hospital. Nor is the case for artificial ventilation much borne out by the experience of French hospitals. At the most recent of Paris hospitals, the Tenon Hospital at Menilmontant, there is a very complete system by which air is forced into the wards by a powerful steam-fan. The experience of the staff was, when I visited the hospital some six years ago, decidedly in favour of open windows as against the artificial system. The same remark was made at the Municipal Hospital of St. Denis, near Paris.

The question I would ask is: Does not the experience of large London hospitals show that it is possible to provide for an ample change of air in the wards by means of open windows, air-gratings at the floor-level, and smoke-flues? If this is so, the justification for a costly system of extraction - flues and heating - surfaces vanishes. Of course it will be understood that I am referring solely to general hospitals, and not to the particular case of the Brompton Hospital, where the conditions are special, and require special treatment.

One point to which Dr. Williams refers in his summary of conclusions—namely, the necessity for maintaining at all times the temperature in the extraction-shaft—cannot, I think, be too strongly emphasised. A stoppage or reversal of the current would inevitably have the effect of loading the ward-air with the dust, which would tend to accumulate on the sides of the extraction-shaft.

